# Physical properties and biological activities of hesperetin and naringenin in complex with methylated β-cyclodextrin

**DOI:** 10.3762/bjoc.11.297

**Published:** 2015-12-29

**Authors:** Waratchada Sangpheak, Jintawee Kicuntod, Roswitha Schuster, Thanyada Rungrotmongkol, Peter Wolschann, Nawee Kungwan, Helmut Viernstein, Monika Mueller, Piamsook Pongsawasdi

**Affiliations:** 1 Structural and Computational Biology Unit, Department of Biochemistry, Faculty of Science, Chulalongkorn University, Bangkok 10330, Thailand; 2 Department of Pharmaceutical Technology and Biopharmaceutics, University of Vienna, Vienna 1090, Austria; 3 Ph.D. Program in Bioinformatics and Computational Biology, Faculty of Science, Chulalongkorn University, Bangkok 10330, Thailand; 4 Institute of Theoretical Chemistry, University of Vienna, Vienna 1090, Austria; 5 Department of Chemistry, Faculty of Science, Chiang Mai University, Chiang Mai, 50200, Thailand; 6 Starch and Cyclodextrin Research Unit, Department of Biochemistry, Faculty of Science, Chulalongkorn University, Bangkok 10330, Thailand

**Keywords:** binding energy, bioactivity, cyclodextrins, hesperetin, naringenin

## Abstract

The aim of this work is to improve physical properties and biological activities of the two flavanones hesperetin and naringenin by complexation with β-cyclodextrin (β-CD) and its methylated derivatives (2,6-di-*O*-methyl-β-cyclodextrin, DM-β-CD and randomly methylated-β-CD, RAMEB). The free energies of inclusion complexes between hesperetin with cyclodextrins (β-CD and DM-β-CD) were theoretically investigated by molecular dynamics simulation. The free energy values obtained suggested a more stable inclusion complex with DM-β-CD. The vdW force is the main guest–host interaction when hesperetin binds with CDs. The phase solubility diagram showed the formation of a soluble complex of A_L_ type, with higher increase in solubility and stability when hesperetin and naringenin were complexed with RAMEB. Solid complexes were prepared by freeze-drying, and the data from differential scanning calorimetry (DSC) confirmed the formation of inclusion complexes. The data obtained by the dissolution method showed that complexation with RAMEB resulted in a better release of both flavanones to aqueous solution. The flavanones-β-CD/DM-β-CD complexes demonstrated a similar or a slight increase in anti-inflammatory activity and cytotoxicity towards three different cancer cell lines. The overall results suggested that solubilities and bioactivities of both flavanones were increased by complexation with methylated β-CDs.

## Introduction

**Figure 1:**
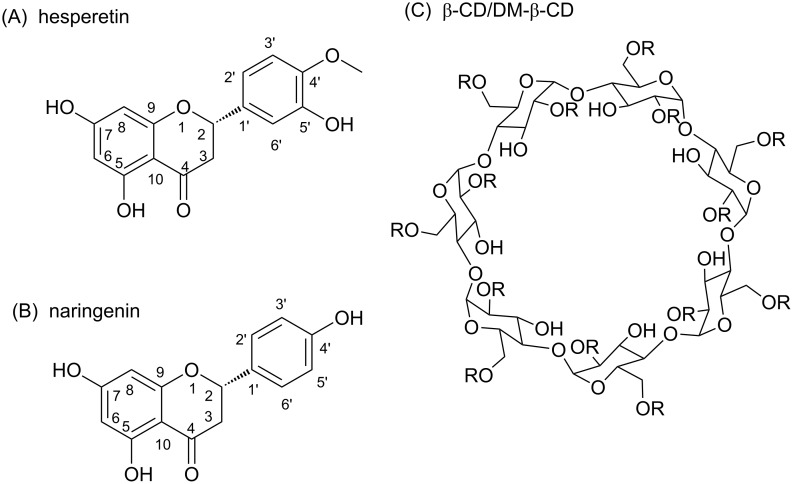
Structures of (A) hesperetin, (B) naringenin and (C) the two cyclodextrins: β-CD and DM-β-CD with the –H and –CH_3_ substitutions on –R groups, respectively. For DM-β-CD, substitution by –CH_3_ is at OH of C2 and C6 while for RAMEB, full substitution is found at OH of C6 but partial at C2 and C3 of each glucose unit.

Flavonoids are secondary metabolites found in fruits, vegetables, grains, roots, bark, stems, flowers, and especially in tea and wine [1]. More than 5,000 naturally occurring different flavonoids have been identified [2], including flavonols, flavones, flavanones, anthocyanidins, isoflavones, and dihydroflavonols. They possess many beneficial biological properties, i.e., free radical scavenging activity [3], cardioprotective action [4], antibacterial and antiviral activities [5]. Two flavonoids in the class of flavanones, hesperetin and naringenin (chemical structures in Figure 1), can be extracted from citrus fruits such as lemon, grapefruit and orange. Several reports on biological effects of hesperetin and naringenin have been found [6], including blood lipid- and cholesterol-lowering effects [7–9], anti-inflammatory [10–13] and anticancer [14–16] activities, improved microcirculation, recovery of venous ulcers, inhibition of chronic venous insufficiency and hemorrhoids, and prevention of post-operative thromboembolism [17]. Hesperetin can inhibit chemically induced mammary [18], urinary bladder [19], colon carcinogenesis in laboratory animals [20–22], and proliferation of breast cancer cells (MCF-7) [23–24]. Naringenin has the ability to hinder a tumor growth on various human cancer cell lines [25], and acts as an inhibitor that blocks basal and insulin-stimulated glucose uptake in breast cancer cells [26]. Additionally, naringenin reduces the incidence of hormone-responsive cancer [27]. In spite of having several benefits, the use of these flavonoids is frequently limited by their low water solubility and stability with a consequence of exerting rather low biological activity.

The natural β-cyclodextrin (β-CD) is a cyclic oligosaccharide consisting of seven D-glucopyranose units linked by α-(1,4)-glycosidic bonds. It contains a highly hydrophobic central cavity and the hydrophilic outer surface. The 2,6-di-*O*-methyl-β-cyclodextrin (DM-β-CD), a commercially available β-CD derivative, is obtained by methylation of the hydroxy groups at C2 and C6 of all glucose units, thus having the degree of substitution of 2.0. While the randomly methylated-β-cyclodextrin (RAMEB) prepared with the average degree of substitution of 1.8, being fully methylated at C6 but partial at C2 and C3 positions (Figure 1) [28]. Cyclodextrin complexation is at present highly relevant to various branches of industry, including pharmaceutical, medicine, cosmetics, food and agriculture [29]. β-CD and its derivatives have been widely used to increase the water solubility, stability, and consequent bioavailability of poorly water soluble drugs [30–32], such as β-CD in complex with piroxicam [33] and etodolac [34] and DM-β-CD in complex with camptothesin [35], chloramphenicol [36] and paclitaxel [37]. DM-β-CD has been found to improve the insulin absorption via pulmonary administration [38], and to stimulate nasal insulin absorption with a reduction of the serious nasal toxicity [39].

In the present work, we aimed to improve physical properties and biological activities of hesperetin and naringenin through complexation with cyclodextrins. Computational tools (molecular dynamics simulation) were adopted to first predict the stability of flavanones/CDs inclusion complexes. Consequently, the experimental phase solubility and dissolution study of flavanones were studied. The formed inclusion complexes were analyzed by DSC. The anti-inflammatory activity of inclusion complexes and the free flavanones were determined by investigating the secretion of certain cytokines in lipopolysaccharide (LPS)-stimulated macrophages. In addition, the cytotoxic activity on CaCo-2, HeLa and MCF-7 carcinoma cell lines of inclusion complexes was also determined.

## Results and Discussion

The binding free energy of inclusion complexes between naringenin with β-CD and DM-β-CD has been previously reported by our group [40]. In the present work, we reported the binding study of hesperetin with the two cyclodextrins. Furthermore, the physical properties of both flavanones when complexation with β-CD and RAMEB were investigated, the RAMEB was used in this part to save the experimental cost and with the anticipation that RAMEB and DM-β-CD should not give much difference to these properties. However, in the third part whereby biological activities were examined, the two flavanones complexing with β-CD and DM-β-CD were used since the different degree of methylation might exert significant effect on the result as previously reported [28].

### Binding free energy of inclusion complexes

Root mean square displacements (RMSDs) for all atoms of the complex, cyclodextrin and hesperetin in respect with those of initial structures (Figure S1, Supporting Information File 1) suggested that the three independent simulations of β-CD (A1–A3) and DM-β-CD (B1–B3) complexes had reached equilibration at 25 ns. The 30 MD snapshots from the last 55 ns of each simulation was selected for binding free energy calculations in accordance with the naringenin/CDs complexes [40].

In this study, we applied a molecular mechanics and continuum solvation method to estimate the binding free energies, or calculate the free energies of molecules in solution (∆*G*_bind_) using the MM-PBSA/GBSA method. The free energy decomposition of each complex in terms of gas phase energy (∆*E*_MM_) including ∆*E*_ele_ and ∆*E*_vdw_ energies, solvation free energy (∆*G*_sol_) and entropic term (*T*∆*S*) is shown in Table 1. It seems that both flavanones interacted with cyclodextrins through van der Waals (vdW) force 2 to 5-fold stronger than electrostatic interaction. The ∆*E*_vdw_ and ∆*E*_ele_ of hesperetin/β-CD and hesperetin/DM-β-CD were −23.58 and −31.30 kcal·mol^−1^ and −9.95 and −5.90 kcal·mol^−1^, respectively (Table 1). For naringenin/β-CD and naringenin/DM-β-CD, the ∆*E*_vdW_ values were −25.69 and −29.71 kcal·mol^−1^ while ∆*E*_ele_ values were −4.09 and −4.73 kcal·mol^−1^, respectively [40] (Supporting Information File 1). Thus, the vdW interaction played an important role in forming the inclusion complex. The obtained information was in good agreement with previous studies in which the hydrophobic interaction was found to be the main driving force for flavanones–CD inclusion complexes [40–41]. For the summation of entropic and solvation terms, both MM/PBSA and MM/GBSA methods predicted that the binding free energy of hesperetin/DM-β-CD was by ≈7.6 kcal·mol^−1^ lower than that of hesperetin/β-CD, and a better binding of naringenin in the cavity of DM-β-CD than in that of β-CD by ≈4.6 kcal·mol^−1^ was suggested [40]. Nevertheless, the MM-PBSA/GBSA binding free energies of the two inclusion complexes might be overestimated due to MM energy. To correct this energy section, the same set of 25 to 80 MD snapshots was carried out by the single point DFT M062X/6-31+g (d,p) calculation in this study. The results of QM/PBSA and QM/GBSA binding free energies were in agreement with MM/PBSA and MM/GBSA energies. The experimental ∆G values showed the same trend with values from molecular dynamics simulation that complexing with DM-β-CD was more effective than with β-CD, and the values obtained were in good agreement with the previous report [42]. These results suggested that both flavanones bind to and interact with DM-β-CD stronger than with β-CD.

**Table 1:** MM-PBSA/GBSA binding free energies (kcal/mol) and energy components between hesperetin and β-CD/DM-β-CD complexes in comparison to experimental values.

energy (kcal/mol)	hesperetin/β-CD	hesperetin/DM-β-CD

∆*E*_ele_	−9.95 ± 0.73	−5.90 ± 0.46
∆*E*_vdW_	−23.58 ± 2.97	−31.30 ± 0.44
∆*E*_MM_	−33.86 ± 3.23	−38.23 ± 0.60
∆*E*_QM_	−28.80 ± 0.03	−31.33 ± 0.03
*T*∆*S*	−12.46 ± 0.37	−11.27 ± 0.07
∆*G*_sol (PBSA)_	15.08 ± 1.78	12.42 ± 2.73
∆*G*_sol (GBSA)_	14.14 ± 1.71	12.79 ± 2.10
∆*G*_MM-PBSA_	−6.32 ± 1.08	−14.54 ± 2.06
∆*G*_MM-GBSA_	−7.25 ± 1.15	−14.17 ± 1.30
∆*G*_QM-PBSA_	−1.26 ± 1.38	−7.64 ± 2.69
∆*G*_QM-GBSA_	−2.20 ± 1.37	−7.27 ± 2.06
*∆G*_experiment_	−3.50	−4.27

### Phase solubility studies

**Figure 2:**
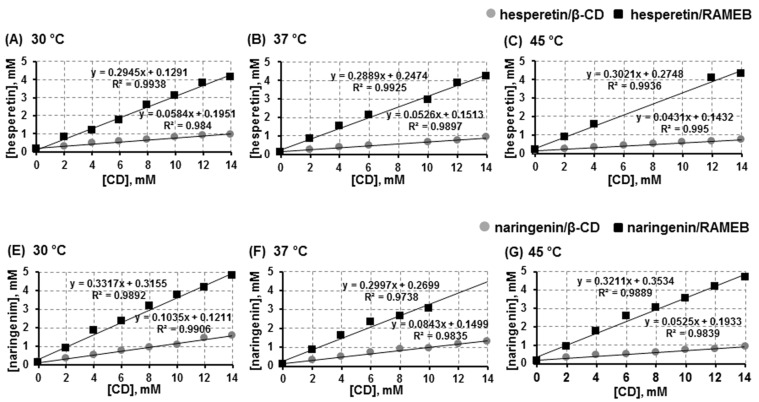
Phase solubility study of hesperetin (upper panel) and naringenin (lower panel) with β-CD or RAMEB in water at 30 °C, 37 °C and 45 °C.

The phase-solubility diagrams for the hesperetin/CD and naringenin/CD complexes at different temperatures are shown in Figure 2. The linear relationship of the plots for all complexes, suggested the typical A_L_-type of the phase solubility profiles with the 1:1 molar ratio of soluble guest and host inclusion complexes [43]. This finding agrees with the previous study which reported complexation between these two flavanones with β-CD [42]. We here found that the aqueous solubility of hesperetin and naringenin were remarkably increased approximately 10 and 40 times by the solubilizing effects of β-CD and RAMEB. Thus, the solubility of both flavanones in the presence of CDs followed the order of RAMEB > β-CD, reflecting an enhancement of binding and solubility of both flavanones.

A previous report by Yang et al. showed a similar result, methylated-β-CDs gave a much better solubilization effect on naringenin than β-CD [31]. The solubility of the naringenin/CD complex was increased to approximately 1.34, 1.60 and 1.52 mg/mL by complexing with β-CD, DM-β-CD and TM-β-CD (trimethyl-β-CD), respectively, while the solubility of free naringenin was 4.38 μg/mL. The methyl substitution plays an important role in balancing the CD water solubility and its complexing ability [44].

It was previously reported that increasing the degree of methylation up to an optimum level improves the CD aqueous solubility, and the binding of guests to CDs is increased by increasing the surface area of binding [41,45–47]. However, beyond the optimum level, the steric hindrances of the host molecule impair CD complexing efficiency or capacity. The summarized data for stability constants (*K*_c_) of the hesperetin/CD and naringenin/CD complexes at different temperatures are shown in Table 2. The stability constant (*K*_c_) was determined from the linear part of the phase solubility diagram assuming a 1:1 complex. The solubility of flavanone in the absence of cyclodextrin can be estimated from the intercept of the plot between the concentration of flavanone and cyclodextrin. It was observed that the stability constant (*K*_c_) of the complexes was affected by temperature, the decrease in temperature resulted in the increase in the *K*_c_ value. These findings are in accordance with the work of Tommasini et al. which reported the stability of hesperetin and naringenin complexes with β-CD at different temperatures in the range of 15–45 °C [42]. The temperature parameter contributes to the strength of the interaction between the host and guest molecules in complex formation. Size matching of the host and guest molecules also dominates complex stability. Our results showed that the binding constants for the complexation of both flavanones with RAMEB were higher than those for β-CD. This suggested that the RAMEB complex was more stable than the β-CD system; the result showed the same trend of advantage of the methyl derivative as in the binding free energy obtained from calculation using the computational method (Table 1). From the result on the binding energy, the vdW energy was about six-fold greater than the electrostatic energy in both of the CDs complexes (Table 1 for hesperetin and Table S1, Supporting Information File 1 for naringenin). This implied that the complex stability was mainly governed by the vdW interaction.

**Table 2:** Stability constants (*K*_c_) of hesperetin/CD complex and naringenin/CD complex at different temperature.

temperature (°C )	*K*_c_ (M^−1^)			
	hesperetin/β-CD	hesperetin/RAMEB	naringenin/β-CD	naringenin/RAMEB

30	339.2	1289.3	425.0	1015.5
37	249.9	1030.5	367.8	999.9
45	195.6	1000.2	203.0	892.7

### DSC analysis

**Figure 3:**
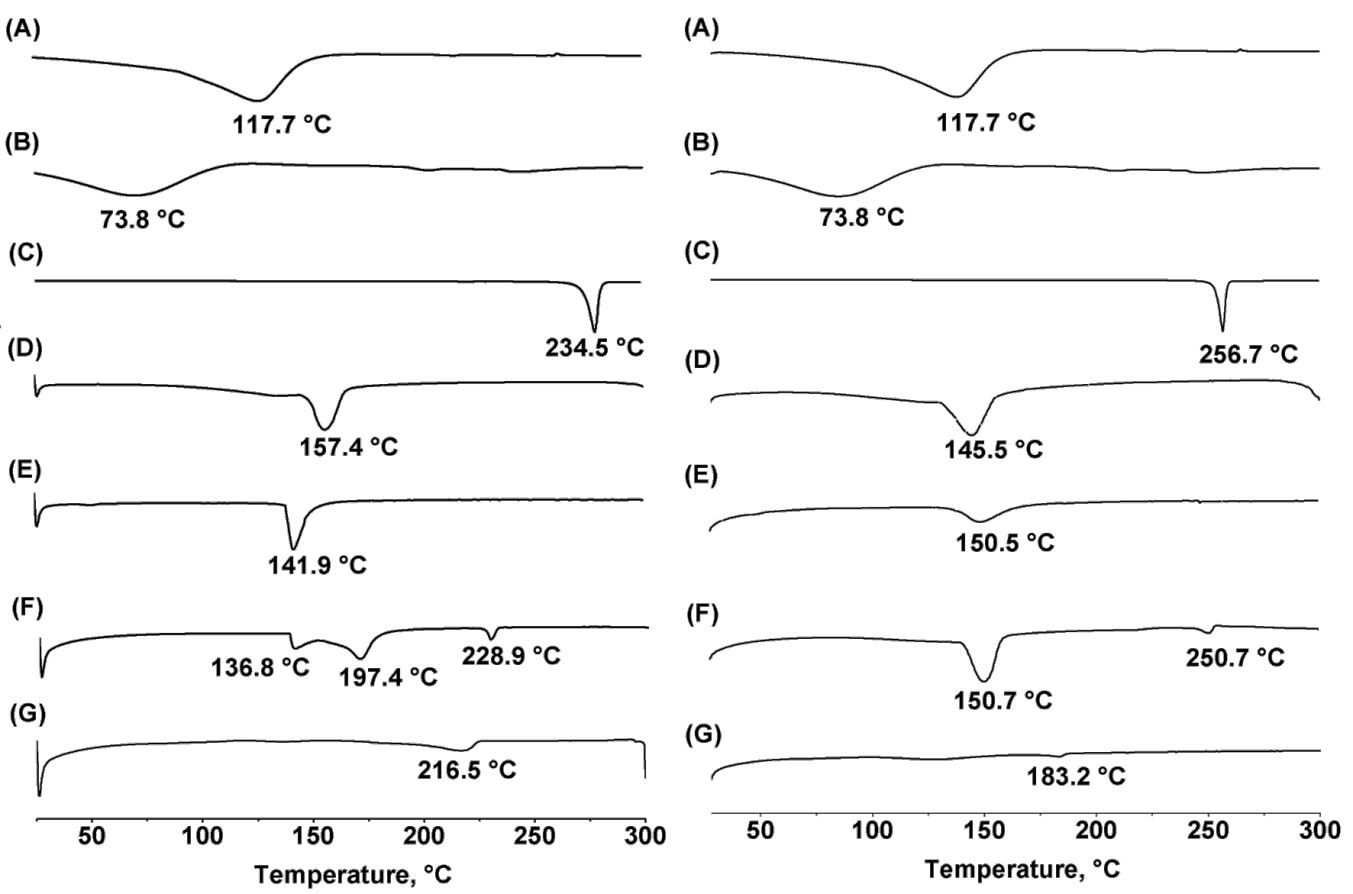
DSC thermograms of hesperetin (left) and naringenin (right) (A) β-CD, (B) RAMEB, (C) Free flavanones, (D and E) flavanones/β-CD and flavanones/RAMEB complexes, respectively, prepared by freeze-drying, (F and G) flavanones/β-CD and flavanones/RAMEB complexes, respectively, prepared by the kneading method.

To investigate the solid inclusion complex obtained by freeze-drying (Figure 3D and E) and kneading methods (Figure 3F and G), DSC measurements were performed. The thermogram revealed information about the thermal properties of the starting free materials (flavanones, β-CD and RAMEB) compared with those inclusion complexes. The melting endothermic peaks are characteristic of each of the free compounds: hesperetin at 234.5 °C (Figure 3C), β-CD at 117.7 °C (Figure 3A), RAMEB at 73.8 °C (Figure 3B) and naringenin at 256.7 °C were determined. In case of the complexes obtained from freeze-drying, the endothermic peaks of free hesperetin and naringenin disappeared, simultaneously with the appearance of a new peak at 157.4 and 141.9 °C for hesperetin/β-CD and hesperetin/RAMEB, respectively (see also Figure 3D and E, left panel). Naringenin/β-CD and naringenin/RAMEB systems showed a new peak at 145.5 and 150.5 °C, respectively (Figure 3D and E, right panel). The complete inclusion complexes were thus observed for the system obtained by freeze-drying. In contrast, the kneading method resulted in a shift to lower temperatures of the flavanones’s melting points in the thermogram of the complexes. In addition, there were peaks that could be ascribed to some flavanones/CD interaction [48]. The result suggested that the kneading method yielded incomplete inclusion complexes.

### Dissolution study

Reports of release studies of several guest/drug molecules, e.g., ampelopsis, simvastatin, ketoconazole and famotidine can be found in literature [49–52]. For dissolution studies of the two flavanones, Tommasini and co-workers reported the dissolution profiles of hesperetin, naringenin and their β-CD complexes in buffer solutions at different pH [42]. They found that the dissolution of the hesperetin complex rapidly increases within 30 minutes at pH 1.5 while the highest amount of dissolved drug was observed at pH 8.0. It was also found that naringenin showed almost the same behavior.

**Figure 4:**
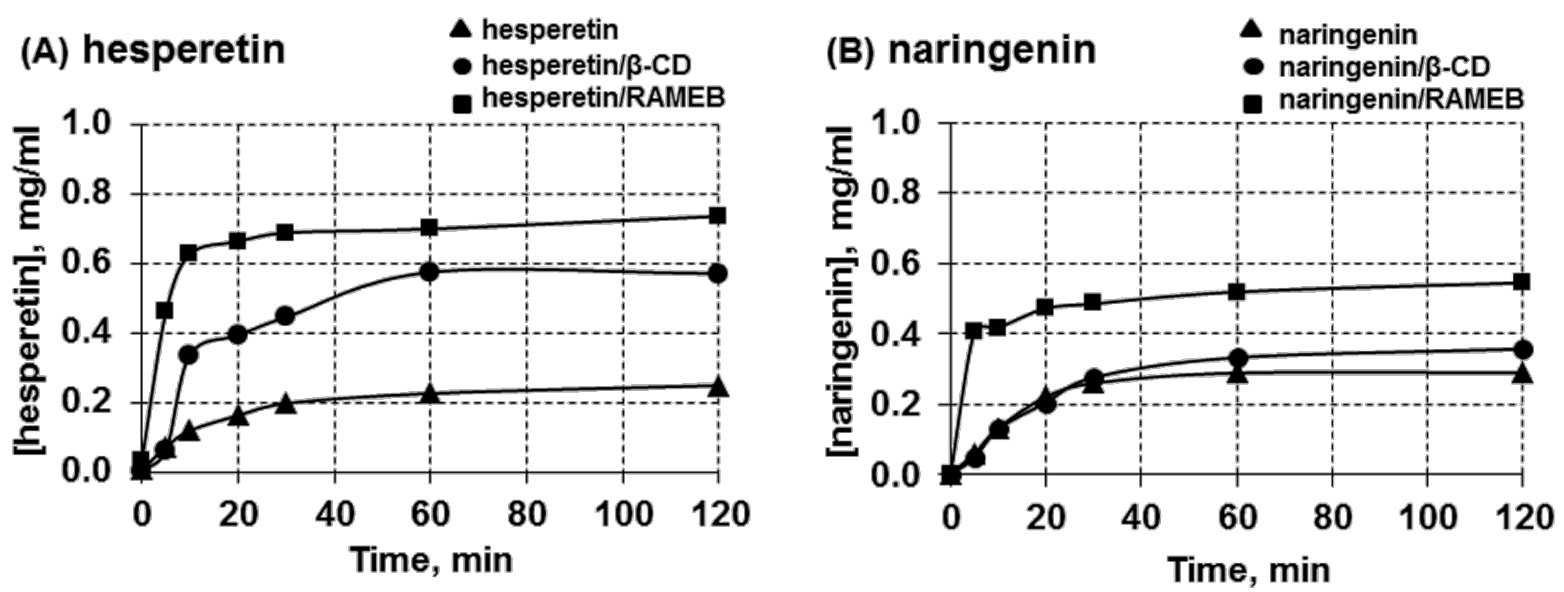
The dissolution diagram at 37 °C in water of the free flavanones and their complexes. (A) hesperetin and (B) naringenin. Complex formation was by freeze-drying.

The dissolution of the solid complexes of the two flavanones and cyclodextrins formed by freeze-drying at a 1:1 molar ratio was determined in this study. The dissolution diagram of the free guests, hesperetin and naringenin, and their inclusion complexes in water at 37 °C are shown in Figure 4. The free form of hesperetin or naringenin exhibited poor dissolution owing to their hydrophobicity. It can be observed that the dissolution of the two flavanones was enhanced significantly when they formed complexes with cyclodextrins. Both free and inclusion complexes of hesperetin and naringenin were dissolved with high rate for the first 10 minutes. When compared the dissolved amounts of free hesperetin, β-CD complex and RAMEB complex after 10 minutes, the amounts of dissolved hesperetin were 0.11, 0.33 and 0.63 mg/mL, respectively. For free naringenin, naringenin/β-CD and naringenin/RAMEB complexes, the amounts of dissolved naringenin were 0.13, 0.13 and 0.42 mg/mL. As effect of the type of the carrier host, it was concluded that the RAMEB system showed a dissolution rate higher than the inclusion complex with β-CD. The dissolved amount of both free and hesperetin complexes increased significantly within 30 minutes. Naringenin showed almost the same behavior, as the greatest solubilization occurs within 30 minutes as well. These results were in good agreement with a previous report [42] in which the dissolution of hesperetin and naringenin complexed with β-CD was studied. The improved dissolution rate observed may be due to the increase in solubility, as well as a decrease in the crystallinity of guest molecules, brought about by complexation with CDs [53].

### Cytokine levels in response to treatment with inclusion complexes of flavanones

**Figure 5:**
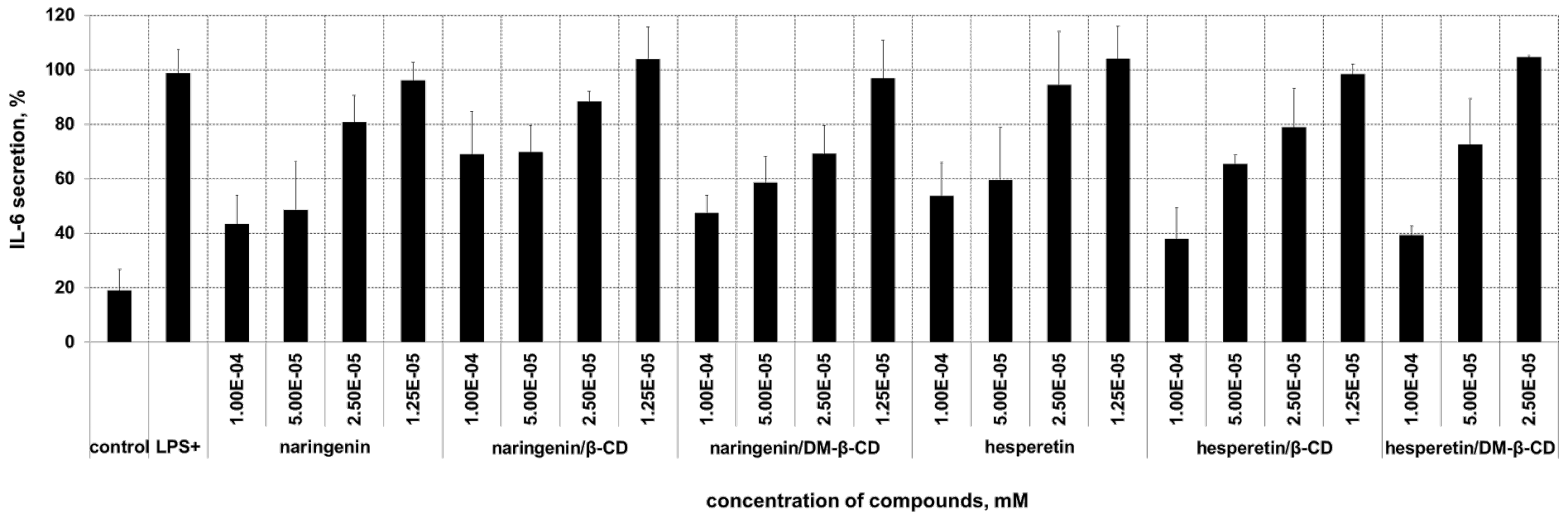
The influence of flavanones and their complexes on IL-6 secretion from LPS-stimulated macrophages.

To investigate the anti-inflammatory effect of the inclusion complexes compared to free compounds, we used LPS-stimulated macrophages for testing the change of interleukin (lL)-6 secretion. The anti-inflammatory effects of flavanones and their inclusion complexes are shown in Figure 5. Secretion of the IL-6 was significantly inhibited by naringenin, naringenin/β-CD and naringenin/DM-β-CD (at least 30%) at 0.1 and 0.05 mM, however, the free and complex form gave a similar result. These findings support previous studies of Mueller and co-workers [54], who showed a comparable effect of naringenin. Bodet and co-workers [55] studied the anti-inflammatory effect of naringenin in macrophages and ex vivo in human whole-blood models. They found that naringenin at high concentrations (25 and 50 µg/mL) significantly reduced the amount of secreted IL-6. In case of hesperetin and their complexes, a reduction of IL-6 secretion by at least 20% at 0.1 and 0.05 mM concentration was found. It is clearly seen that the inclusion complexes of hesperetin at 0.1 mM were more effective than free hesperetin, but only at low concentration, the β-CD complex showed a slightly better effect than the DM-β-CD complex. Complexation of nonpolar drugs with cyclodextrins is known to be beneficial for pharmaceutical research due to the possibility to increase water solubility, stability and bioavailability of those drugs [56–57]. In such a way, the bioavailability of genistein was found to be increased by complexation with CDs [58].

### Cytotoxicity to cancer cell lines

**Figure 6:**
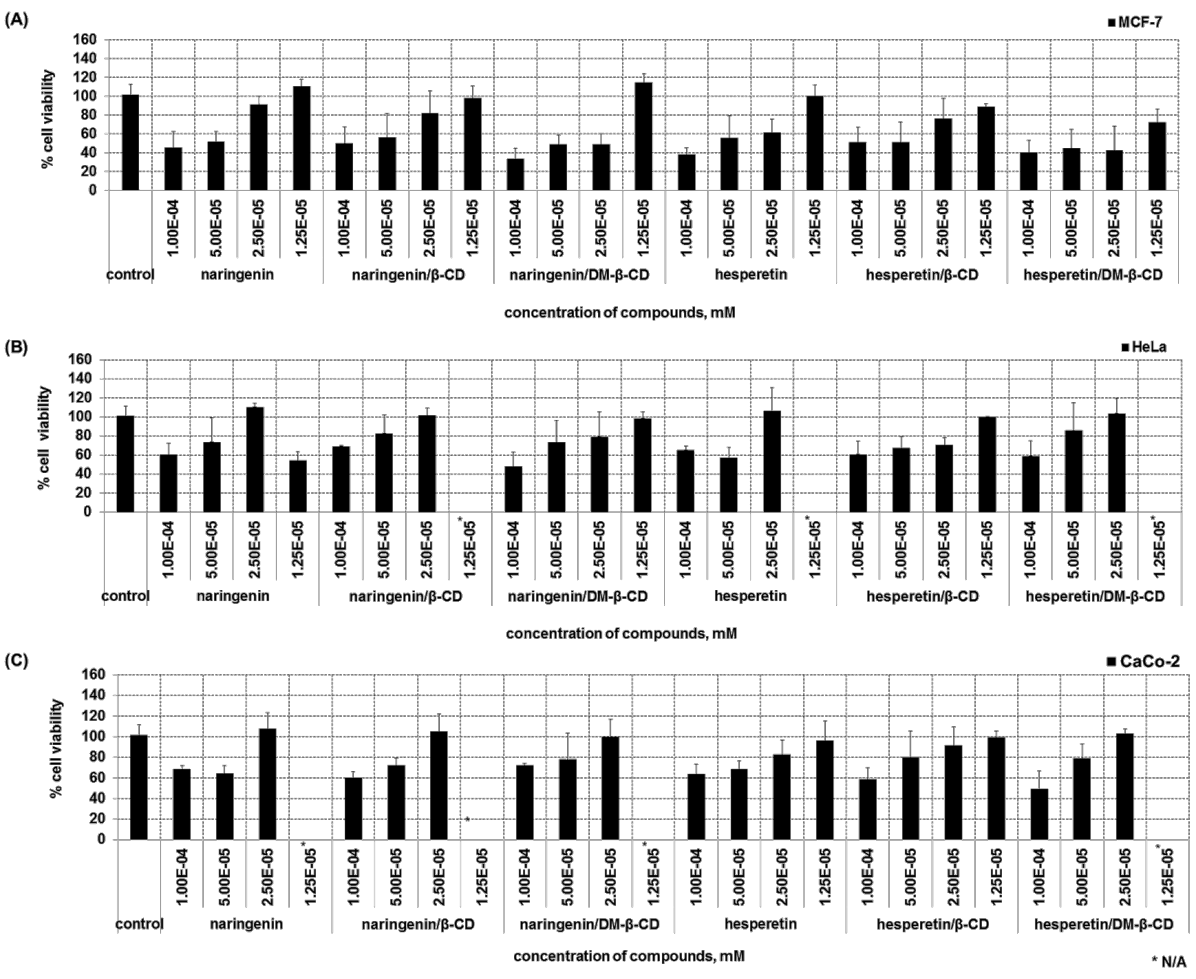
Cytotoxicity of free flavanones and their inclusion complexes on different cancer cell lines.

The cytotoxicity of flavanones and their inclusion complexes against three cancer cell lines (CaCo-2, HeLa and MCF-7) has been determined by MTT assay which measures the metabolic activity and thus viability of cells based on their ability to reduce the tetrazolium substrate to formazan. For the breast cancer cell line (MCF-7), naringenin and their complexes exhibited cytotoxic effects when compared to the control (Figure 6A). This result is consistent with a previous study of Harmon et al. who suggested that naringenin inhibits the proliferation of MCF-7 cells via impaired glucose uptake [26]. Krishnakumar et al. showed the cytotoxic effects between naringenin and naringenin-loaded nanoparticles which exhibited significant cytotoxicity at high concentrations (30, 40 and 50 μg/mL) [59]. At low concentration (0.025 mM), naringenin complexed with DM-β-CD exerted a higher effect on MCF-7 and HeLa cells than free naringenin. However, for CaCo-2 cells, the effect of the naringenin complex was similar to that of the free form (Figure 6C). In case of hesperetin and its complexes, a significant effect on the cell viability on MCF-7, HeLa and CaCo-2 cells was shown at concentrations of 0.1 and 0.5 mM (Figure 6A–C).

## Conclusion

A more stable complexation of hesperetin/DM-β-CD over the β-CD complex was supported by binding free energy calculation, with the vdW force as the main interaction for the inclusion complex. RAMEB gave a higher increase in aqueous solubility and stability of hesperetin and narengenin than β-CD. The stability constant of the inclusion complexes decreased with an increase in temperature. The dissolution of the complexes was increased with a faster dissolution rate than the free flavanones, the RAMEB complex was better dissolved. Naringenin, hesperetin and their inclusion complexes exhibited anti-inflammatory activity as indicated by the reduction of the secretion of the pro-inflammatory IL-6. No significant difference was found in the activity of free and complexed naringenin whereas for hesperetin the anti-inflammatory effect could be slightly increased. The flavanones and their inclusion complexes effectively exerted cytotoxic effects towards cancer cell lines. Complexation mostly leads to a slightly increased effect.

## Experimental

### Materials

Hesperetin was purchased from Cayman Chemicals (Ann Arbor, MI, USA) and β-CD and RAMEB were purchased from Wako Pure Chemical Industries (Osaka, Japan), respectively. Naringenin, DM-β-CD, dimethyl sulfoxide (DMSO), sodium dodecyl sulfate (SDS), thiazolyl blue (MTT), compounds for phosphate buffer saline (PBS) and lipopolysaccharides (LPS) from *Escherichia coli* O111:B4 were obtained from Sigma-Aldrich (Darmstadt, Germany). Dulbecco’s Modified Eagle’s Medium (DMEM) was obtained from Life Technologies (Carlsbad, CA, USA). Anti-Mouse IL-6 was purchased from eBioscience Inc. (San Diego, CA, USA). Human colon cancer (Caco-2) cells, breast cancer (MCF-7) cells and human cervical carcinoma (HeLa) cells were obtained from the American Type Cell Culture Collection (ATCC), USA.

### Methods

#### Binding free energy calculation

**Figure 7:**
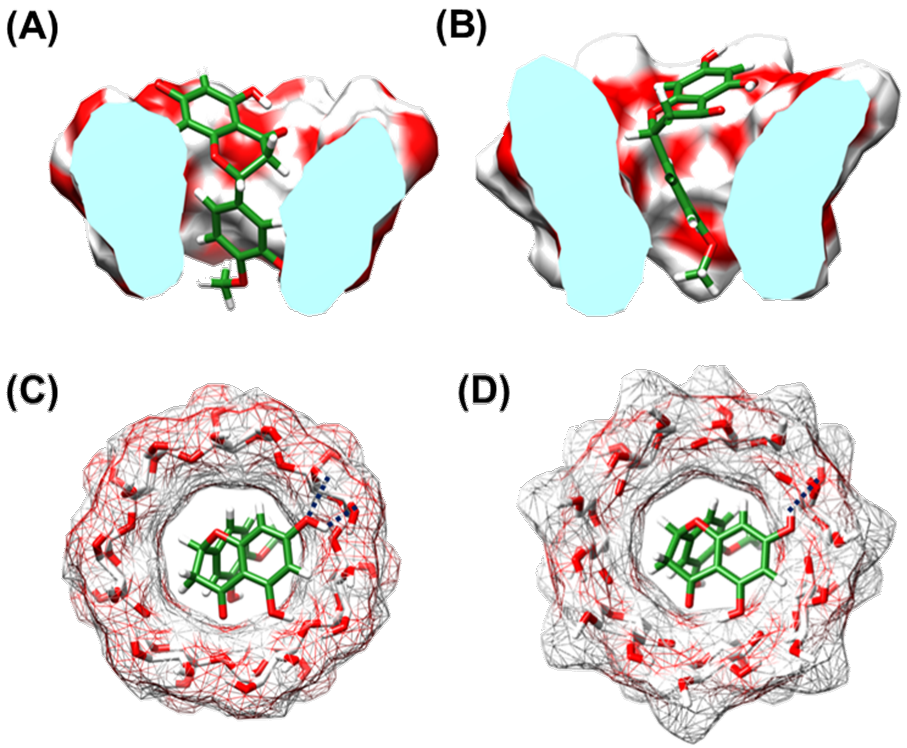
The docked orientations of hesperetin in the hydrophobic cavity of (A) β-CD and (B) DM-β-CD and top views of the CD hydrophobic cavity and H-bond showing (C) phenyl ring inserted into β-CD’s cavity (D) phenyl ring inserted into DM-β-CD’s cavity.

Molecular dynamics (MD) simulations with periodic boundary condition were performed on the three best docked structures of the hesperetin/CDs complexes (Figure 7) similar to our previous studies on naringenin/CDs complexes [40,60] using the Amber 12 software package [61]. Note that the docked structures were resulted from the CDOCKER module implemented in Accelrys Discovery Studio 2.5 (Accelrys, Inc.). The Glycam-06 bimolecular force field was applied on the cyclodextrins, while the partial atomic charges and parameters of hesperetin were developed by the standard procedure [32,62–63]. Using a truncated octahedral box, the SPC water molecules were solvated with a spacing distance of 12 Å from the complex surface. Each system was heated up to 298 K within 100 ps and followed by the 80 ns simulation with NPT ensemble at the same temperature, 1 atm and time step of 2 fs. The non-bonded interaction was truncated within a 12 Å cutoff distance. The particle-mesh of Ewald’s method [64] was used for adequate treatment of the long-range electrostatic interactions with 12 Å cutoff. All bond lengths involving hydrogen atoms were constrained by SHAKE [32]. The coordinates were recorded every 500 steps for analysis. The MM- and QM-PBSA/GBSA calculations were conducted to estimate the binding free energy of the inclusion complex [40,60]. For QM calculation, the single point M06-2X/6-31+G** level of theory including the empirical dispersion correction energy [46] was treated on the same set of structures of inclusion complex, cyclodextrin and hesperetin using Gaussian09 program package [65].

#### Phase solubility studies

Phase solubility studies were carried out according to Higuchi and Connors [43]. Excess amount of flavanones were added to a series of 0–14 mM concentration of cyclodextrin (β-CD and RAMEB) solutions. The mixtures were shaken at 30 ± 0.5, 37 ± 0.5 and 45 ± 0.5 °C for 72 hours in a water bath shaker. After equilibrium, the samples were centrifuged at 12,000 rpm for 15 minutes; then the solubility was determined by measuring the absorbance at 256 nm using a DU650 UV visible spectrophotometer. The apparent stability constant *K*_c_ was calculated from the phase solubility diagram by means of Equation 1.

[1]
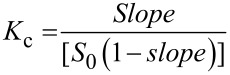


#### Inclusion complexation preparation

The inclusion complex of flavanones/CDs (β-CD and RAMEB) in a 1:1 molar ratio was prepared by freeze-drying. Each compound was accurately weighed, then dissolved in distilled water (10 mL) and sealed in a flask. The mixture was stirred magnetically at room temperature for 24 hours. Subsequently, the solution was filtered through a 0.45 µm pore size filter, frozen overnight and then lyophilized (LYO-LAB, Lyophilization Systems, Inc USA) over the period of 24 hours. The dried powder was stored in desiccators for further use.

#### Differential scanning calorimetry (DSC) analysis

The differential scanning calorimetry (DSC, Netzsch, 204 F1 Phoenix) was used for recording DSC thermograms of the free hesperetin and naringenin and the inclusion complexes with β-CD and RAMEB. The thermal behavior was studied by heating samples (2–5 mg) in closed aluminum crimped pans at a rate of 10 °C min^−1^ between a temperature range of 25 to 250 °C for both hesperetin and naringenin [42].

#### Dissolution study

Dissolution studies of all samples were carried out in 20 mL of distilled water. Hesperetin or naringenin and its solid complexes with β-CD and RAMEB (5 mg) were added in distilled water, and shaken at 37 °C, 1 mL was withdrawn for analysis of the hesperetin or naringenin content at different time intervals (0, 5, 10, 20, 30, 60 and 120 minutes). The sample was diluted to appropriate concentration, and analyzed for absorbance at 286 nm by high-performance liquid chromatography (HPLC, Waters 600, USA). The dissolution studies were performed in triplicate.

#### Determination of biological activity of flavanones

**Preparation of free compound and their complexes:** Stock solutions of 100 mM of free compounds were prepared in water. Inclusion complexes of hesperetin and naringenin with β-CD and DM-β-CD were prepared by freeze drying and diluted with distilled water to a final concentration of 100 mM.

**Anti-inflammatory assay:** Macrophages (RAW 264.7, ATCC) were used to examine the effect of free compounds and their inclusion complexes on inflammation as described previously [59]. Cells were seeded at a density of 2 × 10^6^ cells per well in 24 well plates, and incubated at 37 °C for 24 hours. After that, samples were added to obtain a final concentration of 0.1, 0.05 and 0.025 mM preincubated for 3 hours and then a final concentration of 1 µg/mL of LPS was added. The cells were incubated for a further 24 hours at 37 °C. On the following day, the media supernatant was removed, centrifuged and stored at −20 °C prior to analysis by ELISA. The negative control experiments were cells untreated with LPS and the positive control experiments were cells incubated with LPS only. An enzyme linked immunosorbent assay (ELISA) was used for the determination of the concentration of secreted IL-6 of the cells in the supernatant. All incubation steps were performed at room temperature according to the manufacturer´s protocol (eBiosciences, Santa Clara, CA, USA). The absorbance was measured at 450 nm and corrected by background absorbance at 570 nm, using a Genios Pro microplate reader (Tecan, Crailsheim, Germany). After removing the supernatant for ELISA analysis, the cells were incubated with MTT (0.5 mg/mL) for 3 hours at 37 °C. Then, the cells were lysed using 10% SDS in 0.01 N HCl. To monitor cell viability, secreted cytokine levels were measured at the suitable cell density. The absorbance at 570 nm was measured for the formazan product and corrected by the absorbance at a reference wavelength of 690 nm, using a Genios Pro microplate reader. Positive control was performed using cells incubated with LPS only and was set at 100%. All experiments were performed in three separate measurements and the results are shown as mean with error bars representing the standard deviation.

**Cytotoxicity towards cancer cell lines:** An MTT assay was performed to determine the cell viability and thus the cytotoxicity of the test compounds towards three different cancer cell lines (HeLa, CaCo-2, MCF-7). Cells were seeded into 96-well plates at a density of 2 × 10^6^ cells/mL and incubated for 24 hours under normal culture conditions. On the next day, test substances were added and the cells were incubated for another 24 hours. Then, 10 µL MTT solution was added, incubated for 2 hours and cells were lysed. The absorption was measured at 570 nm with correction for background at 690 nm as described above, using an Infinite M200 microplate reader. The amount of cells of the positive control (cells only incubated with DMEM) was defined as 100%. The results from the test substances were calculated as a percentage of the positive control. All experiments were separately performed in triplicate and the results are shown as mean with error bars representing the standard deviation.

## Supporting Information

File 1 Additional data.
