# Supplementary material for: Physical properties and biological activities of hesperetin and naringenin in complex with methylated β-cyclodextrin
Source: Beilstein J Org Chem. 2015 Dec 29;11:2763–73. doi: 10.3762/bjoc.11.297 (PMC4734351; doi:10.3762/bjoc.11.297)
Supplement: File 1 — Additional data. [file Beilstein_J_Org_Chem-11-2763-s001.pdf]

Supporting Information

for

Physical properties and biological activities of hesperetin and naringenin in complex with methylated β-cyclodextrin

Waratchada Sangpheak[1], Jintawee Kicuntod[1], Roswitha Schuster[2], Thanyada Rungrotmongkol[1,3], Peter Wolschann[1,2,4], Nawee Kungwan[5], Helmut Viernstein[2], Monika Mueller*[,2], Piamsook Pongsawasdi[*,6§]

Address: [1]Structural and Computational Biology Unit, Department of Biochemistry, Faculty of Science, Chulalongkorn University, Bangkok 10330, Thailand, [2]Department of Pharmaceutical Technology and Biopharmaceutics, University of Vienna, Vienna 1090, Austria, [3]Ph.D. Program in Bioinformatics and Computational Biology, Faculty of Science, Chulalongkorn University, Bangkok 10330, Thailand, [4]Institute of Theoretical Chemistry, University of Vienna, Vienna 1090, Austria, [5]Department of Chemistry, Faculty of Science, Chiang Mai University, Chiang Mai, 50200, Thailand and [6]Starch and Cyclodextrin Research Unit, Department of Biochemistry, Faculty of Science, Chulalongkorn University, Bangkok 10330, Thailand

Email address: Monika Mueller - monika.mueller@univie.ac.at; Piamsook Pongsawasdi - piamsook.p@chula.ac.th

[*]Corresponding author

[§]Tel: +66 2 2185423; Fax: + 66 2 2185418

Additional data

System stability

The RMSDs of the hesperetin with β-CD and DM-β-CD are shown in Figure S1. The RMSD plots of the 9 simulated inclusion complexes (black) suggested that all systems had reached equilibrium at 25 ns and thus the obtained results from the last 55 ns were mainly extracted for binding free energy.

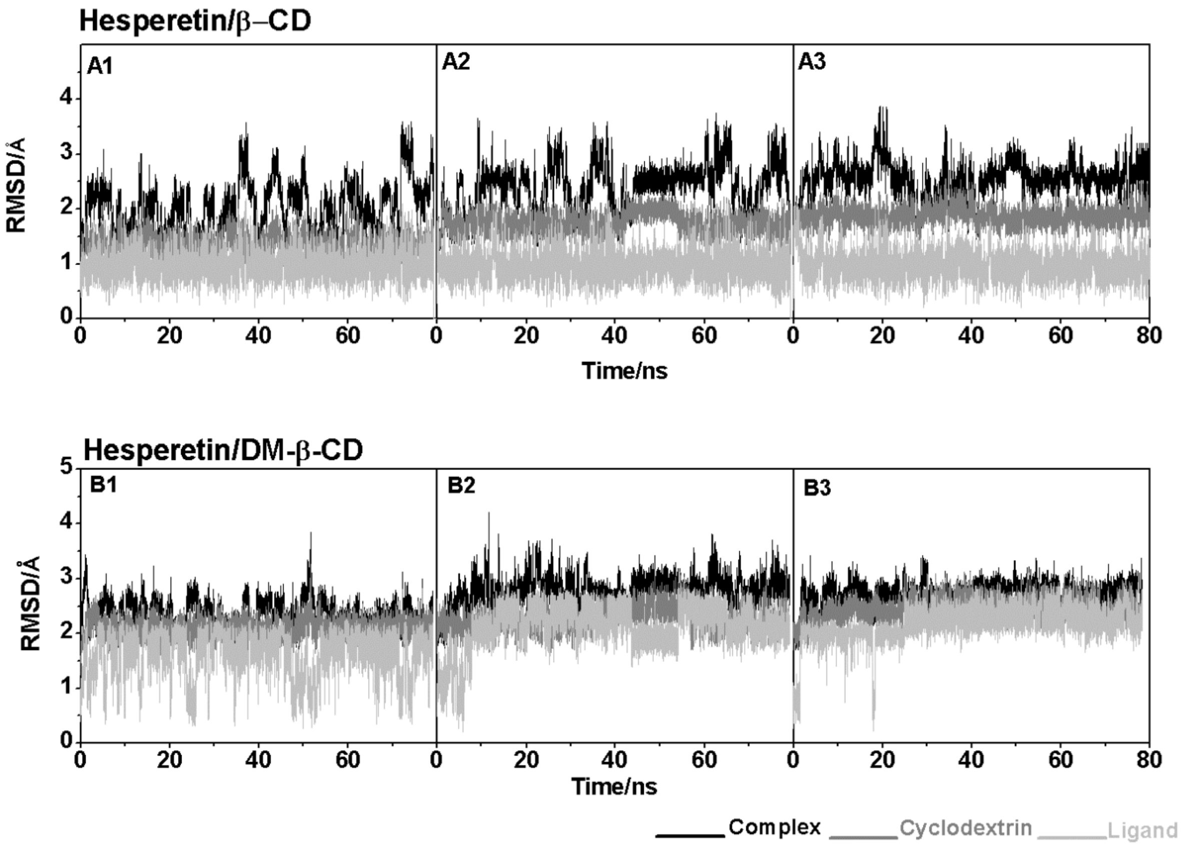

Figure S1: RMSD plots all atoms for the simulated systems of the hesperetin in complex with β-CD and DM-β-CD from the three different docked conformations: (A1–A3) for complex with β-CD, (B1–B3) for complex with DM-β-CD.

Binding energy analysis

MM-PBSA binding free energies of the naringenin and β-CD/DM-β-CD complexes are presented in Table S1. It can be seen that the binding free energy of the naringenin in complex with DM-β-CD was considerably more stable than those with β-CD.

Table S1: MM-PBSA/GBSA binding free energies (kcal/mol) and energy components between naringenin and βCD/DMβCD complexes in comparison to experimental values.

Energy (kcal/mol)	naringenin/β-CD	naringenin/DM-β-CD
ΔE_{ele}	-4.09±2.89	-4.73±3.65
ΔE_{vdw}	-25.69±2.85	-29.71±2.61
ΔE_{MM}	-29.78±4.32	-34.43±3.54
ΔE_{QM}	-23.51±0.01	-27.05±0.03
$T\Delta S$	-11.70±1.31	-14.1±5.82
$\Delta G_{Sol\ (PBSA)}$	11.65±2.12	11.64±1.97
$\Delta G_{Sol\ (GBSA)}$	10.64±2.19	9.28±1.63
$\Delta G_{MM/PBSA}$	-5.89±1.25	-8.70±2.57
$\Delta G_{MM/GBSA}$	-7.44±1.46	-11.06±2.69
$\Delta G_{QM/PBSA}$	-0.16±0.08	-1.31±1.42
$\Delta G_{QM/GBSA}$	-1.17±0.04	-3.67±1.75
$\Delta G_{experiment}$	-3.64	-4.27
